# Cameron lesion with severe iron deficiency anemia and review of literature

**DOI:** 10.22088/cjim.13.3.639

**Published:** 2022

**Authors:** Abhishek Singhai, Rishabh Bose, Piyush Manoria

**Affiliations:** 1 Department of Medicine, All India Institute of Medical Sciences, Bhopal, India; 2 Manoria Hospital and research centre, Bhopal, India

**Keywords:** Hiatal hernia, Endoscopy, Bleeding, Anemia

## Abstract

**Background:**

Iron deficiency is the most common cause of anemia in many developing countries including India. Inadequate iron in diet, poor iron absorption, excessive bleeding, or chronic blood loss in the stool/ urine may be the cause. Cameron lesions are mucosa injuries of gastric body or fundus in the background of hiatal hernia.

**Case Presentation:**

Here we describe a case report of a 50-year-old female who presented to hospital with pain in abdomen. During laboratory workup she had severe anemia due to iron deficiency. Esophagogastroduodenoscopy revealed a large hiatal hernia with a superficial ulcer present in the hiatal pouch, the GE junction being 35 cm from the incisors. So, a hiatus hernia with a Cameron ulcer was identified as the culprit of iron deficiency anemia.

**Conclusion:**

The diagnosis of a Cameron lesion is difficult and sometimes ignored. In patients with anemia/bleeding, thorough surveillance of all stomach folds is essential, especially if a significant hiatal hernia is present.

Iron deficiency is the most common cause of anemia in many developing countries including India. Globally, 50% of anemia are due to iron deficiency and nearly a million people die due to iron deficiency anemia annually worldwide.^1^ Inadequate iron in diet, poor iron absorption, excessive bleeding, or chronic blood loss in the stool/ urine may be the cause. Cameron lesions are mucosa injuries of gastric body or fundus in the background of hiatal hernia, particularly when it is large (> 5 cm). Diagnosis of a Cameron lesion is very challenging and often overlooked. Endoscopy is investigation of choice for diagnosis of Cameron lesions but these lesions are commonly missed also. In patients with anemia/bleeding, careful antegrade and retrograde viewing of the region, as well as perpendicular views of the hernial neck and adjacent mucosa, are essential, especially if a big hiatal hernia is present. Here, we present a case of iron deficiency anemia caused by a Cameron lesion. 

## Case Presentation

A 50-year-old female presented to the Medicine OPD of a tertiary care centre with complaints of pain in abdomen mainly over the epigastric region (which used to be exacerbated with food intake) for the last 5 years but has increased in severity for the last 3 months. She also complained of fever on and off which she has not measured, constipation, generalized body ache and gradually progressive generalized body swelling since last 3 months. There was no history of associated hematemesis, melena, nausea, vomiting, loose stool or burning micturition. 

Patient was non-diabetic and non-hypertensive. She had menopause 10 years back and prior to that she had normal menstrual history. Patient had addiction of areca nut chewing for 35 years. At the time of presentation, patient had pulse rate of 98/minute, blood pressure 89/56 mmHg. She was afebrile. She was pale and had koilonychia. Her abdomen was soft on palpation. Investigations revealed substantial anemia (hemoglobin 2.4 gm/dl, hematocrit 10.3%) and microcytic hypochromic RBCs. 

**Figure 1:**
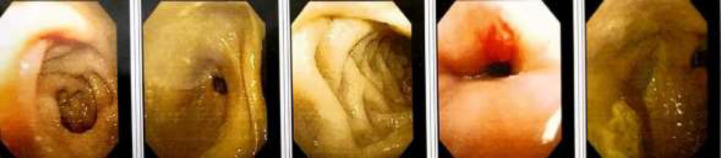
Upper GI endoscopy showing a hiatal hernia with a small clean based ulcer

Other parameters were serum iron 4 mcg/dl, iron saturation of 0.81 %, serum TIBC 493.12 mcg/dl, all indicative of severe iron deficiency anemia. Two units of packed red blood corpuscles were transfused to the patient. This was followed by pantoprazole and iron sucrose administration. A gastrointestinal bleeding source was considered as the most likely cause of the iron-deficiency. Esophagogastroduodenoscopy revealed a large hiatal hernia with a superficial clean based ulcer present in the hiatal pouch, the GE junction being 35 cm from the incisors. (Figure 1) So, a hiatus hernia with a Cameron ulcer was identified as the culprit of iron deficiency anemia. 

Patient improved after 5 days treatment. Repeat investigations showed increase in hemoglobin (6.4 gm/dl) and hematocrit (20.6%). On discharge, the patient was advised to continue pantoprazole, domperidone and lifestyle modifications for the management of hiatus hernia.

## Discussion

Cameron lesions are injuries to the mucosa of the stomach body or fundus that arise in hiatal hernia patients. Large hiatal hernia (> 5 cm) is a risk factor for a Cameron lesion. Mechanical damage caused by diaphragmatic constriction during respiratory excursions and acid injury are two possible etiologies. Local ischemia in gastric body folds due to mechanical stress applied by diaphragmatic pressure on the occluded hiatal sac, is the major pathogenesis (2).

Back diffusion of gastric acid on the injured location also contributes to the lesion's development and/or persistence. These lesions may appear as a single or many linear erosions on endoscopy, with erythematous borders (3). Because Cameron lesions can be difficult to see, they may be missed during endoscopy. Sometimes, minor lesion may heal by itself that leads to possible endoscopic underreporting. Cameron lesions vary in prevalence depending on the size of the hiatal hernia, ranging from 1% in small to 13% in large.

Furthermore, larger hernias (> 5 cm) are more frequently related with iron deficiency anemia than smaller hernias (3 cm) (4). Cameron lesions can cause blood loss through either persistent, microscopic GI bleeding or overt hemorrhage, even if anemia is not present. In individuals with iron deficiency anemia/obscure bleeding, the prevalence of Cameron lesions ranges from 1.9 percent to 9.2 percent, while it is less common (0.2 percent) in those with overt GI bleeding (5, 6). PubMed was used to conduct a systematic review of the literature. From January 2000 to July 15, 2020, the search was confined to the medical terms ‘Cameron lesions and ‘stomach’. A total of 58 studies were identified. Anemia was a presenting symptom in 62% of patients; overt bleeding (hematemesis and/or melena) in 36% of patients, and hypovolemic shock (2%) and vomiting (1%) were also found in these studies. Furthermore, 31 (69%) of the 45 patients with the presented data had already had one or more upper endoscopies before being diagnosed. The majority of them were managed with proton pump inhibitors (PPIs) and iron supplements, although 40 (31%) required blood transfusions, 12 (9%) required endoscopic hemostasis, and 37 (29%) required surgical intervention for either hiatal hernia repair (36 cases) or urgent hemostasis (one patient) (3-10). Gastric lesion healing and anemia recovery were achieved in the majority of cases after PPI therapy and iron supplementation, while rebleeding occurred in four cases and one patient died.

Notably, nearly two-thirds of patients finally diagnosed with Cameron lesions had already had one (or more) upper GI endoscopy. Cameron lesions are an important entity to consider in case of obscure gastrointestinal bleeding. Reaching to diagnosis in our case was difficult, and it was only accomplished after thorough diagnostic procedures, indicating that these lesions could be missed.

In individuals with iron deficient anemia, the Cameron lesion remains a difficult and often misdiagnosed condition. In patients with anemia/bleeding, thorough surveillance of all stomach folds is essential, especially if a significant hiatal hernia is present.

PPI therapy is the cornerstone of treatment. When diagnosed with Cameron lesions, however, more than half of the patients were already on regular PPI medication. As a result, a Cameroon lesion is likely to require either long-term treatment with a sufficient dose of PPI or a surgical technique for hiatal hernia correction.
